# Deep learning system improved detection efficacy of fetal intracranial malformations in a randomized controlled trial

**DOI:** 10.1038/s41746-023-00932-6

**Published:** 2023-10-13

**Authors:** Meifang Lin, Qian Zhou, Ting Lei, Ning Shang, Qiao zheng, Xiaoqin He, Nan Wang, Hongning Xie

**Affiliations:** 1 Department of Ultrasonic Medicine, Fetal Medical Center, First Affiliated Hospital of Sun Yat-sen University, Guangzhou Guangdong, China; 2 Department of Medical Statistics, Clinical Trials Unit, First Affiliated Hospital of Sun Yat-sen University, Guangzhou, Guangdong, China and Zhongshan School of Medicine, Sun Yat-sen University, Guangzhou Guangdong, China; 3 Department of Ultrasound, Guangdong Women and Children Hospital, Guangzhou Guangdong, China; 4 Department of Ultrasound, Women and Children’s Hospital affiliated to Xiamen University, Xiamen Fujian, China; 5 Guangzhou Aiyunji Information Technology co., Ltd, Guangzhou Guangdong, China

**Keywords:** Randomized controlled trials, Neuronal development

## Abstract

Congenital malformations of the central nervous system are among the most common major congenital malformations. Deep learning systems have come to the fore in prenatal diagnosis of congenital malformation, but the impact of deep learning-assisted detection of congenital intracranial malformations from fetal neurosonographic images has not been evaluated. Here we report a three-way crossover, randomized control trial (Trial Registration: ChiCTR2100048233) that assesses the efficacy of a deep learning system, the Prenatal Ultrasound Diagnosis Artificial Intelligence Conduct System (PAICS), in assisting fetal intracranial malformation detection. A total of 709 fetal neurosonographic images/videos are read interactively by 36 sonologists of different expertise levels in three reading modes: unassisted mode (without PAICS assistance), concurrent mode (using PAICS at the beginning of the assessment) and second mode (using PAICS after a fully unaided interpretation). Aided by PAICS, the average accuracy of the unassisted mode (73%) is increased by the concurrent mode (80%; *P* < 0.001) and the second mode (82%; *P* < 0.001). Correspondingly, the AUC is increased from 0.85 to 0.89 and to 0.90, respectively (*P* < 0.001 for all). The median read time per data is slightly increased in concurrent mode but substantially prolonged in the second mode, from 6 s to 7 s and to 11 s (*P* < 0.001 for all). In conclusion, PAICS in both concurrent and second modes has the potential to improve sonologists’ performance in detecting fetal intracranial malformations from neurosonographic data. PAICS is more efficient when used concurrently for all readers.

## Introduction

Artificial intelligence (AI), especially deep learning (DL), is increasingly used in image medicine and health care^1–4^. Much of the work about DL has focused on the retrospective evaluation of model performance on ground-truth-labelled validation datasets, and few studies have gone a step forward to evaluate the impact of DL assistance on sonologist diagnostic performance, let alone explore the suitable assistant modes of DL in clinical diagnosis^5–8^. In a recent study, we developed a DL system named PAICS (the Prenatal ultrasound diagnosis Artificial Intelligence Conduct System) that is capable of identifying 9 types of fetal intracranial abnormalities and normal image patterns. The performance of PAICS in off-line images and videos was comparable to that of the experts and due to its high accuracy and efficiency, the system could serve as a central nervous system (CNS) malformation screening tool^9^. PAICS has received extensive attention from medical professionals and was acclaimed as a major advancement of AI in the obstetrical ultrasound field^10^. To further develop the PAICS, we focused on the following questions: first, can the PAICS be integrated into clinical practice with efficacy? Second, what assisted method is more suitable when PAICS is applied?

Two assistant reading modes are widely employed in radiology in computer-aided detection (CAD): the concurrent mode and the second mode^11–15^. In the concurrent mode, CAD is applied at the start of the assessment, whereas in the second mode, CAD is applied after a full, unassisted reading is completed by the reader. Neither mode is perfect. For example, the concurrent application of CAD may reduce readers’ vigilance and sensitivity^16^, while the second mode is very time-consuming.

In the present study, a multi-reader and crossover randomized controlled trial (RCT) test was performed in which 36 sonologists are recruited to read fetal neurosonographic images/videos without PAICS assistance and with PAICS in two aided modes. We aim to evaluate the efficacy of PAICS in assisting fetal intracranial malformation diagnosis and compares the auxiliary diagnosis methods for the system.

Our work demonstrates that the two image/video reading modes powered by the PAICS deep learning system can significantly increase the accuracy of the classification of CNS malformation. The system shows great potential in improving the performance of sonologists in detecting fetal intracranial malformations.

## Results

### The constitution of fetal neurosonographic images/videos in the reading test

**Fig. 1:**
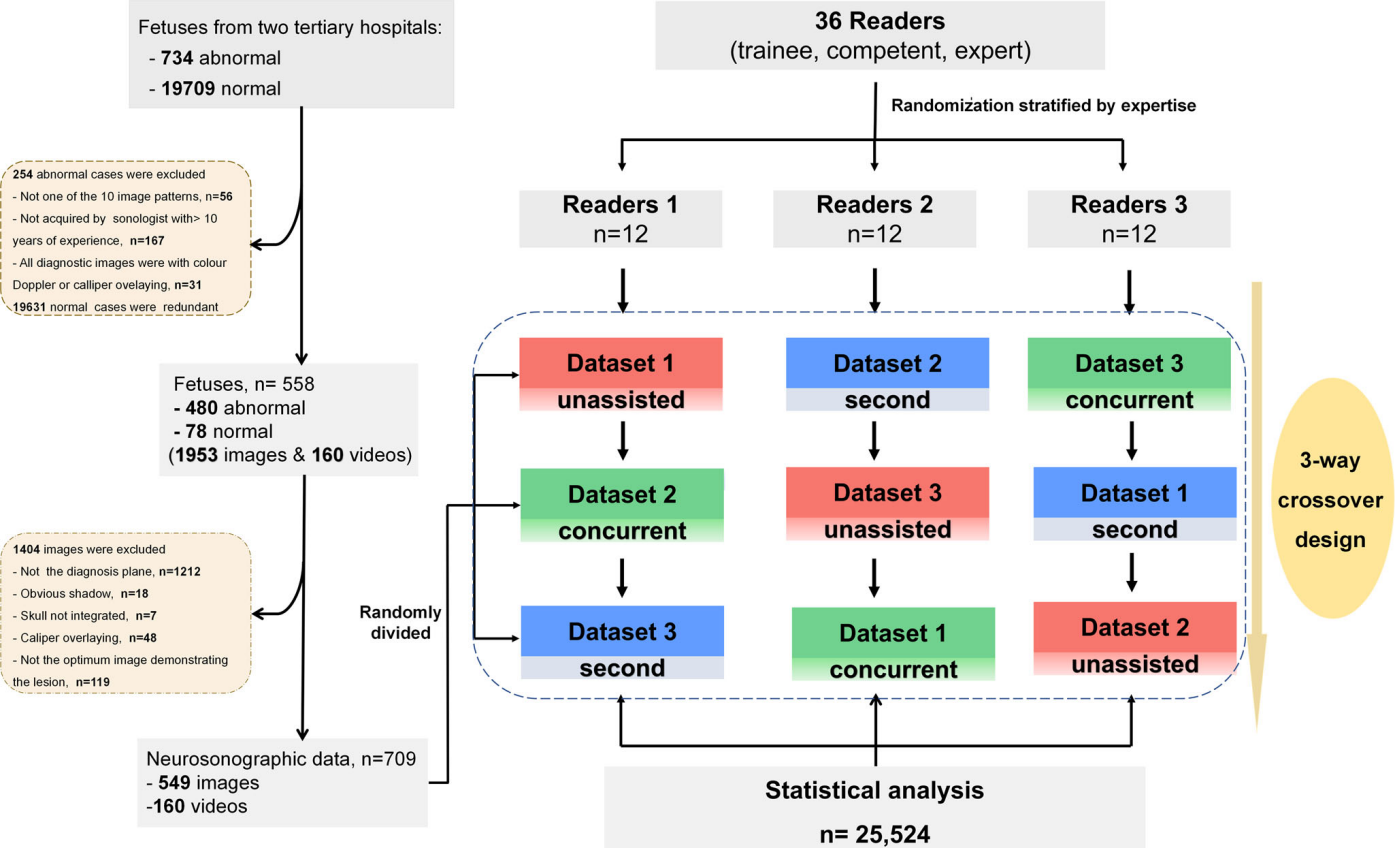
Flowchart of patient selection and randomization for the reading study. A total of 709 original images/videos (549 images, 160 videos) met the inclusion criteria and were eventually included in the study. Eligible neurosonographic data were randomly grouped into three datasets and read interactively by three groups of readers in three reading modes.

During the research period, 734 fetuses with abnormal intracranial findings and 19,709 normal fetuses were scanned, among which 254 fetuses with abnormal findings and 19,631 normal fetuses were excluded because the images were either unqualified or redundant. Finally, a total of 709 original images/videos (549 images, 160 videos) from 558 fetuses met the inclusion criteria and were included (Table 1, Fig. 1 and Supplementary Table 1). According to the trial design, the sum of the sample size expanded from 709 to 25,524 after being read by 36 sonologists with three reading modes, corresponding to 8508 images/videos per reading mode. The eligible neurosonographic data were randomly grouped into three datasets, where the distribution of ten types of patterns did not differ among the three datasets both in image sets (Chi-squared test, *P* = 1.000) and in video sets (Fisher’s Exact Test, *P* = 0.991), and there were no significant differences in the baseline characteristics among the three datasets.

**Table 1:** The constitution of neurosonographic images/videos included in the test.

Patterns	Fetuses	Number of images and videos by datasets			Number of images and videos read by 36 sonologists		
		Dataset 1 (images/videos)	Dataset 2 (images/videos)	Dataset 3 (images/ videos)	Unassisted mode	Concurrent mode	Second mode
Nonvisualization of CSP	84	33 (25/8)	36 (25/11)	35 (25/10)	1248	1248	1248
Nonvisualization of SP	47	25 (15/10)	20 (14/6)	21 (14/7)	792	792	792
Crescent-shaped single ventricle	59	20 (19/1)	21 (20/1)	21 (20/1)	744	744	744
Mild VM	40	19 (14/5)	21 (15/6)	23 (15/8)	756	756	756
Severe VM	70	27 (24/3)	25 (22/3)	23 (20/3)	900	900	900
Nonintraventricular cyst	25	17 (12/5)	15 (12/3)	14 (12/2)	552	552	552
Intraventricular cyst	48	20 (16/4)	22 (15/7)	22 (14/8)	768	768	768
Open fourth ventricle	49	18 (15/3)	17 (15/2)	18 (16/2)	636	636	636
Megacisterna magna	58	27 (22/5)	28 (23/5)	22 (20/2)	924	924	924
Normal	78	33 (23/10)	33 (24/9)	33 (23/10)	1188	1188	1188
Total	558	239 (185/54)	238 (185/53)	232 (179/53)	8508	8508	8508

The number of datasets for each reading mode is 12 times the original data. *CSP* Cavum septi pellucidi, *SP* Septum pellucidum, *VM* Ventriculomegaly.

### Improvements in the sonologist’s performance by PAICS

**Fig. 2:**
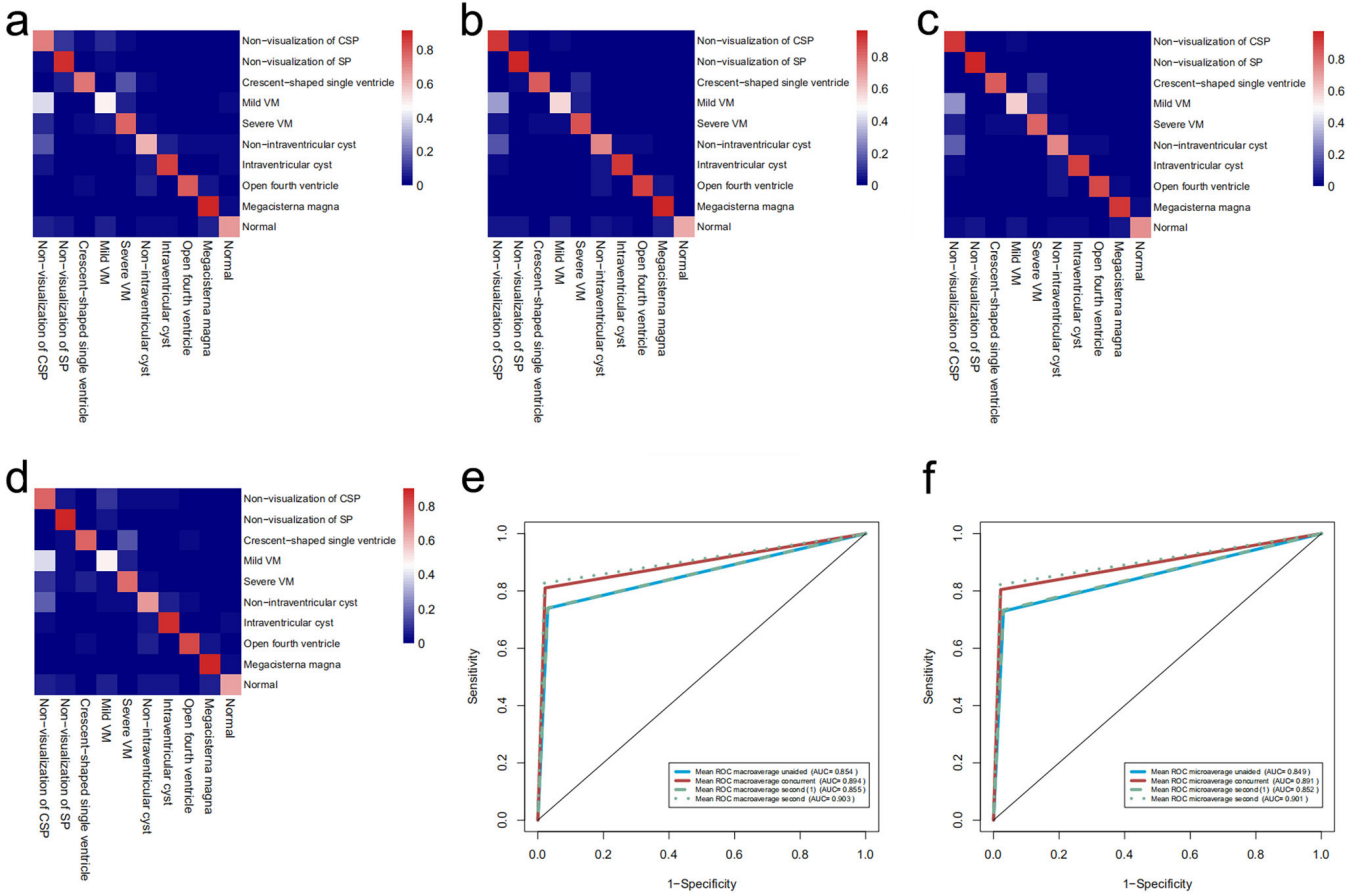
Sonologists performance in recognizing ten fetal neurosonographic image patterns with three reading modes. Confusion matrix of unassisted mode (**a**); concurrent mode (**b**); second mode (**c**); second mode (1), the performance before AI assistance in second mode reading (**d**); Macroaverage AUCs of the three reading modes (**e**); Microaverage AUCs of the three reading modes (**f**). When assisted with PAICS in both concurrent mode and second mode, performance improved significantly, and performance in unassisted mode showed no difference from that in second mode (1). CSP Cavum septi pellucidi, SP Septum pellucidum, VM Ventriculomegaly.

When assisted with PAICS both in concurrent mode and in second mode, the average ACC of all sonologists was increased from 0.73 (95% CI: 0.72, 0.74) in unassisted mode to 0.80 (95% CI: 0.80, 0.81; Chi-square test, *P* < 0.001) in concurrent mode and to 0.82 (95% CI: 0.81, 0.83, Chi-square test, *P* < 0.001) in second mode, yielding increments of 0.08 (95% CI: 0.06, 0.09) and 0.09 (95% CI: 0.08, 0.11) for concurrent and second assistance modes, respectively. Correspondingly, the sensitivity was increased from 0.74 (95% CI: 0.44, 1.00) to 0.81 (95% CI: 0.57, 1.00) and to 0.83 (95% CI: 0.60, 1.00) when assessed by macroaverage and from 0.73 (95% CI: 0.72, 0.74) to 0.81 (95% CI: 0.80, 0.81) and to 0.82 (95% CI: 0.81, 0.83) when assessed by microaverage (Paired t-test for macro- and Chi-square test for microaverage sensitivity, all *P* < 0.001). The AUCs improved from 0.85 to 0.89 and to 0.90 when assessed with macroaverage and microaverage, respectively (Paired t test for macro- and Delong’s test for microaverage AUC, all *P* < 0.001) (Tables 2, 3, Fig. 2).

**Table 2:** The average ACC for sonologists of three levels of expertise in detecting ten fetal neurosonographic image patterns with three reading modes.

	Unassisted mode	Concurrent mode	Second Mode	Comparisons					
				Concurrent vs. Unassisted	*P*	Second vs. Unassisted	*P*	Concurrent vs. Second	*P*
All	0.73 (0.72, 0.74)	0.80 (0.80, 0.81)	0.82 (0.81, 0.83)	0.08 (0.06, 0.09)	< 0.001	0.09 (0.08, 0.11)	< 0.001	0.02 (0.01, 0.03)	0.000
Expert	0.76 (0.75, 0.78)	0.81 (0.80, 0.83)	0.84 (0.82, 0.85)	0.05 (0.03, 0.07)	< 0.001	0.07 (0.05, 0.01)	< 0.001	0.02 (0.01, 0.05)	0.020
Competent	0.74 (0.72, 0.753)	0.82 (0.81, 0.83)	0.84 (0.83, 0.85)	0.08 (0.06, 0.10)	< 0.001	0.10 (0.08, 0.13)	< 0.001	0.021 (0.00, 0.04)	0.040
Training	0.69 (0.67, 0.71)	0.78 (0.77, 0.80)	0.79 (0.77, 0.80)	0.09 (0.07, 0.12)	< 0.001	0.10 (0.08, 0.12)	< 0.001	0.01 (-0.02, 0.03)	0.580

Values in parentheses are 95% Confidence Interval. *ACC* Accuracy. ACCs between each two groups were compared by chi-square test for proportions. Multiple comparisons were corrected by Bonferroni method.

**Table 3:** The multiclass classification performance of sonologists in detecting ten fetal neurosonographic image patterns with three reading modes.

		Unassisted mode	Concurrent mode	Second mode	Second mode (1)	*P*-values for multiple comparisons			
						Concurrent vs. Unassisted	Second vs. Unassisted	Concurrent vs. Second	Second (1) vs. Unassisted
AUC	Macro	0.85 (0.70, 1.00)	0.89 (0.78, 1.00)	0.90 (0.79, 1.00)	0.86 (0.71, 1.00)	< 0.001	< 0.001	0.006	0.800
	Micro	0.85 (0.85, 0.86)	0.89 (0.89, 0.90)	0.90 (0.90, 0.91)	0.85 (0.85, 0.86)	< 0.001	< 0.001	0.001	0.520
SEN	Macro	0.74 (0.44, 1.00)	0.81 (0.57, 1.00)	0.83 (0.60, 1.00)	0.74 (0.44, 1.00)	0.001	< 0.001	0.022	0.880
	Micro	0.73 (0.72, 0.74)	0.81 (0.80, 0.81)	0.82 (0.81, 0.83)	0.73 (0.72, 0 .74)	< 0.001	< 0.001	0.004	0.570
SPE	Macro	0.97 (0.92, 1.00)	0.98 (0.93, 1.00)	0.98 (0.94, 1.00)	0.97 (0.92, 1.00)	0.004	0.001	0.320	0.880
	Micro	0.97 (0.96, 0.97)	0.98 (0.97, 0.98)	0.98 (0.97, 0.98)	0.97 (0.96, 0.97)	0.140	0.005	< 0.001	0.001
ACC	Macro	0.95 (0.89, 1.00)	0.96 (0.91, 1.00)	0.96 (0.92, 1.00)	0.95 (0.89, 1.00)	0.001	< 0.001	0.040	0.620
	Micro	0.95 (0.94, 0.95)	0.96 (0.96, 0.97)	0.96 (0.96, 0.97)	0.95 (0.94, 0.95)	0.460	< 0.001	< 0.001	0.040
FK	Macro	0.70	0.78	0.80	0.70	N/C	N/C	N/C	N/C
	Micro	0.70	0.79	0.80	0.71	N/C	N/C	N/C	N/C

Values in parentheses are 95% Confidence Interval. Second mode (1), the results before AI assistance in second reading mode. *AUC* Area under the receiver operating characteristic curve, *SEN* Sensitivity, *SPE* Specificity, *ACC* Accuracy. *N/C* did not compare. Macro and Micro AUCs between each two groups were compared by paired *t*-test and Delong’s test, respectively. Macro and Micro SENs, SPEs, ACCs between each two groups were compared by paired *t*-test and chi-square test for proportions, respectively. Multiple comparisons were corrected by Bonferroni method.

Among the ten image patterns, the performance on 8 patterns (except for megacisterna magna [Delong’s test, *P* = 0.770] and open fourth ventricle [Delong’s test, *P* = 0.030) in concurrent mode and all patterns in the second mode were improved compared with that of the unassisted mode in terms of AUC (Delong’s test, *P* < 0.010) (Table 4, Fig. 2).

**Table 4:** Comparison of AUCs in the recognition of ten fetal neurosonographic image patterns between three assisted modes.

AUC of ten patterns recognition	Unassisted mode	Concurrent mode	Second mode	Second mode (1)	*P* values of multiple comparisons			
					Concurrent vs. Unassisted	Second vs. Unassisted	Concurrent vs. Second	Second (1) vs. Unassisted
Nonvisualization of CSP	0.68 (0.67, 0.69)	0.77 (0.76, 0.78)	0.78 (0.77, 0.80)	0.69 (0.68, 0.71)	< 0.001	< 0.001	0.175	0.229
Nonvisualization of SP	0.84 (0.82, 0.85)	0.90 (0.88, 0.91)	0.92 (0.90, 0.93)	0.87 (0.85, 0.88)	< 0.001	< 0.001	0.066	0.015
Crescent-shaped single ventricle	0.91 (0.89, 0.92)	0.95 (0.94, 0.96)	0.95 (0.94, 0.96)	0.90 (0.88, 0.91)	< 0.001	< 0.001	0.669	0.304
Mild VM	0.81 (0.80, 0.83)	0.86 (0.85, 0.88)	0.87 (0.86, 0.88)	0.80 (0.78, 0.82)	< 0.001	< 0.001	0.379	0.360
Severe VM	0.85 (0.83, 0.86)	0.89 (0.88, 0.91)	0.90 (0.89, 0.91)	0.85 (0.83, 0.86)	< 0.001	< 0.001	0.599	0.749
Nonintraventricular cyst	0.80 (0.78, 0.82)	0.84 (0.81, 0.86)	0.84 (0.82, 0.86)	0.79 (0.77, 0.81)	0.005	0.005	0.988	0.622
Intraventricular cyst	0.91 (0.89, 0.92)	0.93 (0.92, 0.94)	0.94 (0.93, 0.96)	0.90 (0.88, 0.91)	0.008	< 0.001	0.257	0.383
Open fourth ventricle	0.93 (0.92, 0.95)	0.95 (0.94, 0.96)	0.96 (0.95, 0.97)	0.94 (0.93, 0.95)	0.030	0.004	0.507	0.483
Megacisterna magna	0.91 (0.89, 0.92)	0.91 (0.90, 0.92)	0.94 (0.93, 0.95)	0.90 (0.89, 0.91)	0.770	< 0.001	0.001	0.661
Normal	0.92 (0.91, 0.93)	0.94 (0.94, 0.95)	0.95 (0.94, 0.96)	0.92 (0.92, 0.93)	< 0.001	< 0.001	0.371	0.301

*CSP* Cavum septi pellucidi, *SP* Septum pellucidum, *VM* Ventriculomegaly; second mode (1), the results before AI assistance in second reading mode. AUCs between each two groups were compared by Delong’s test. Multiple comparisons were corrected by Bonferroni method.

Further comparison between the performance of the concurrent and the second reading modes (Tables 2,3, Fig. 2) showed a 2% improvement in average ACC and approximately 1% increments in macro- and microaverage AUCs in the second reading mode. However, these differences did not reach the 3% target in our study design. Additionally, we compared the performance before assistance in the second reading mode with that of the unassisted mode (Table 3, Fig. 2), and no significant difference was found in terms of macro- and micro AUCs (Paired *t*-test for macro- and Delong’s test for microaverage AUC, *P* = 0.520, *P* = 0.800, respectively).

### The performance was improved by PAICS for sonologists of all three levels of expertise

**Fig. 3:**
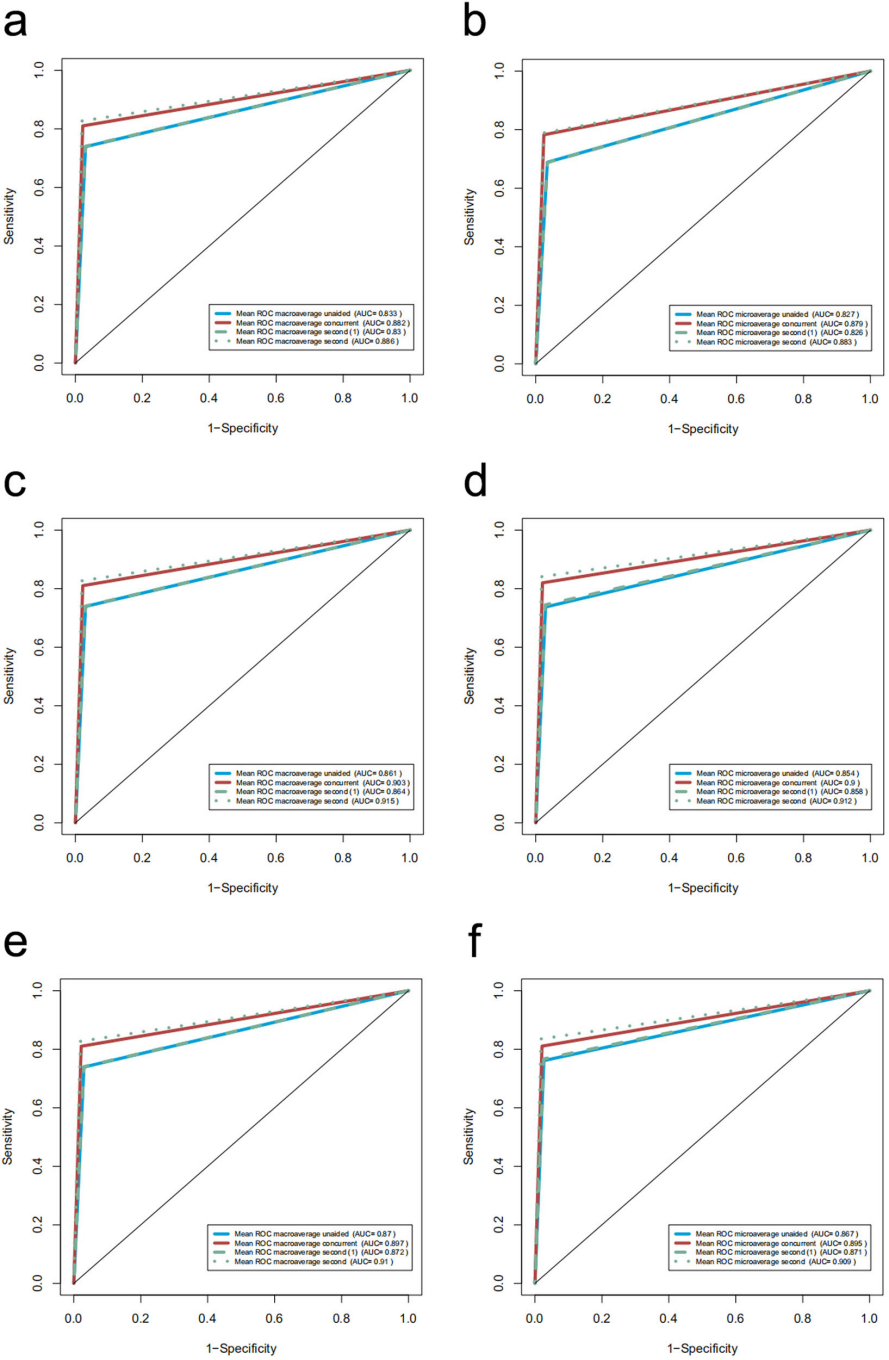
The performance of sonologists of three expertise levels in recognizing ten fetal neurosonographic image patterns with three reading modes. Macroaverage and microaverage AUCs for training (**a**, **b**); for competent sonologists (**c**, **d**); for experts (**e**, **f**). When supported with PAICS in both concurrent mode and second mode, performance improved significantly for all three levels of sonologists.

The performance of experts, competent sonologists, and trainees were improved when assisted by PAICS both as a concurrent reader and as a second reader (Table 2, Fig. 3 and Supplementary Tables 2, 3), manifested in the improvements of average ACC and macro- and micro AUCs comparisons (Delong’s test *P* < 0.0167). When we compared the performance between the two DL-aided modes, it was found that neither mode showed significant advantages to the other mode for these three levels of sonologists, with all of the average ACC differences less than 3% (Table 2).

### The time comparison between three reading modes

**Fig. 4:**
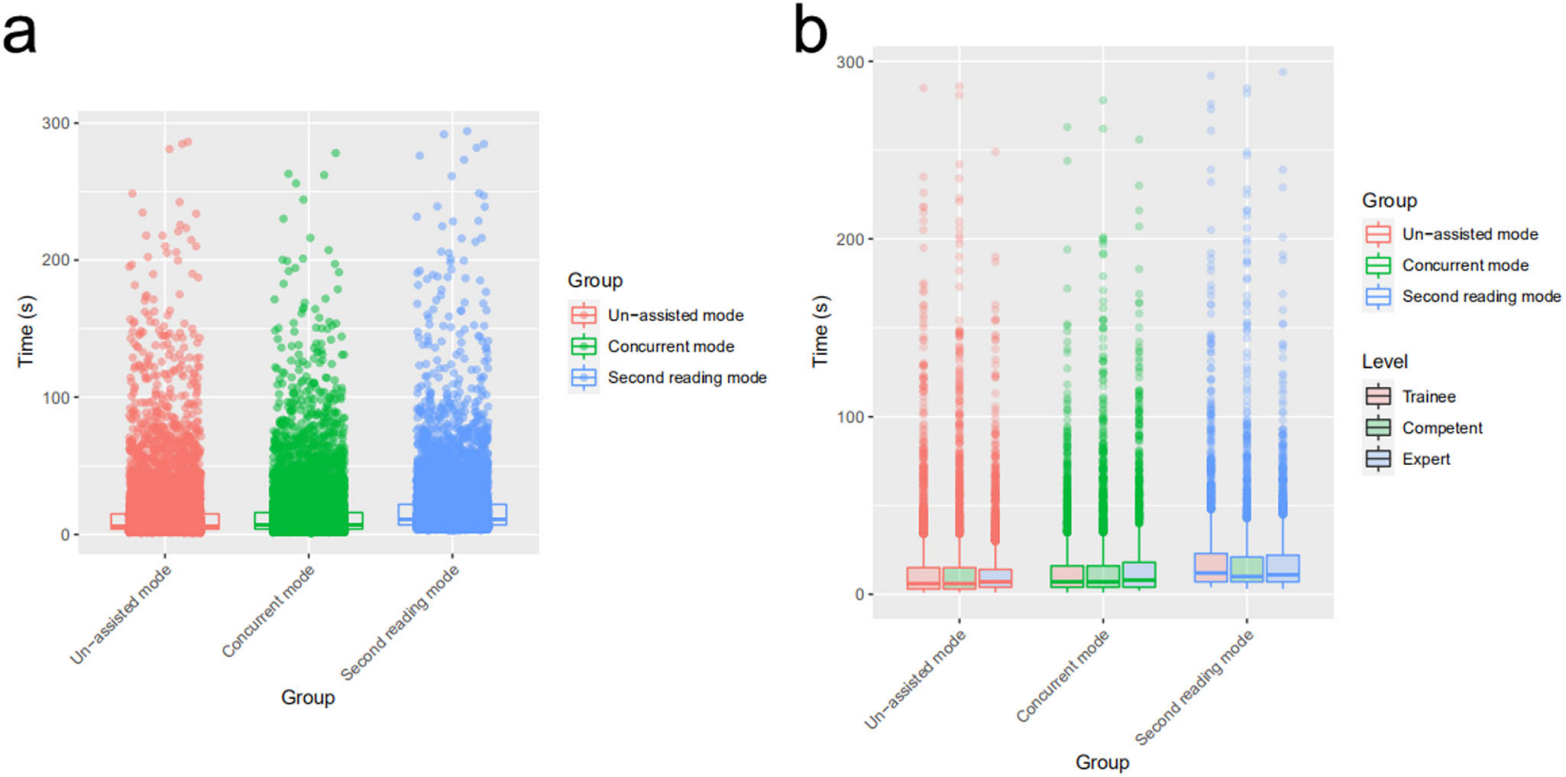
Time consumption for sonologists of three levels of expertise in recognizing fetal neurosonographic image patterns with three reading modes. Compared with the median per-image reading time in unassisted mode, a slight increment in concurrent mode was observed, whereas the time was significantly prolonged in second mode for all sonologists (**a**); and sonologists of three expertise levels, respectively (**b**). The upper and lower bounds of the box refer to the 25th and 75th percentiles, and the line intersection in the box refers to the median. The dots outside of whiskers refer to outliers.

Compared with the median per-image reading time of 6 s in unassisted mode (IQR: [4, 15]), a slight increment in concurrent mode was observed (median (IQR): 7 [4, 16] seconds, Wilcoxon rank sum test, *P* < 0.001), whereas the time was significantly prolonged in second mode (median (IQR): 11 [7, 22] seconds, Wilcoxon rank sum test, *P* < 0.001). The time comparisons for sonologists with different levels of expertise were consistent with those of the average sonologists (Fig. 4 and Supplementary Table 4).

### Questionnaire on sonologists’ subjective evaluation of the efficacy of PAICS

The results indicated that all readers regarded PAICS as helpful in assisting the diagnosis, and the extent varied with scores ranging from 50 to 100 (50 [*n* = 1], 60 [*n* = 2], 70 [*n* = 13], 80 [*n* = 17], 90 [*n* = 2], and 100 [*n* = 1]), resulting in a median score of 80 [IQR: 70–80]. A total of 91.7% (*n* = 33) credited the benefit of PAICS assistance to lesion localization, and 77.8% (*n* = 28) attributed the benefit to the diagnosis that PAICS made, among which 69% (*n* = 25) ascribed it to both. For the preference modes, 61% (*n* = 22) favoured the concurrent mode, and 39% (*n* = 14) preferred the second mode (McNemar’s test, *P* = 0.182). As shown in Supplementary Table 5, there was no significant difference in the preferred modes between sonologists of the three levels (Chi-square test, *P* > 0.05 for all).

## Discussion

Before the application of AI in clinical practice, rigorous randomized controlled trials (RCTs), as the gold standard design, were important to prove the effectiveness of AI-aided diagnosis^17–21^. RCTs involving DL-assisted diagnosis were sparse and mostly limited to colonoscopy diagnosis^5,6,21^. The present research is the preliminary RCT evaluation of the efficacy of DL in obstetrics with respect to the diagnosis of congenital malformations. The PAICS is proven to be valuable in assisting sonologists in recognizing abnormal fetal intracranial patterns, achieving improvements of 8% and 9% in average ACC when using concurrent and second modes, respectively. Concurrent reading and second reading are usually utilized in CAD-assisted reading in other fields, such as breast cancer diagnosis on automated breast US^11,13^, polyp detection in CT colonography^14,15^, and lung nodule identification on multidetector CT^12^. This is the first implementation in obstetric AI assistant scenarios. In the current study, when compared to the unassisted mode, the performance of megacisterna magna and open fourth ventricle diagnosis showed no significant difference with concurrent modes, whereas the second reading mode did; the value of ACC of the concurrent mode was slightly lower than that of the second mode. However, the difference (2%) did not reach the preset value (3%). Therefore, our study indicated there was no significant difference between the accuracies of these two modes, which was consistent with other studies^11,12^. Notably, the concurrent mode took much less time than the second mode and thus was considered more efficient^11,12^. Therefore, through this well-designed RCT reading test, balancing between performance and time consumption, we regarded concurrent mode as a more efficient assisted mode and will be more suitable to use in obstetric ultrasound, which opens more ways for clinical application of DL.

The improvements by PAICS assistance might be ascribed to the localization of the lesion and the final diagnosis that PAICS provided. As shown in the questionnaire of readers’ subjective evaluations, 91.7% (*n* = 33) credited the benefit of PAICS assistance to lesion localization, and 77.8% (*n* = 28) attributed it to the diagnosis that PAICS made. Malformations of the CNS are among the most common major congenital malformations^22^. The incidence of craniocerebral anomalies was estimated to be 9.8/10000 births in Europe^23^. Given an estimated 17.8% annual rate of childbirth in a total of 7,921,784,993 world population^24^, many craniocerebral anomalies can be detected prenatally with the assistance of DL, enabling earlier intervention and appropriate management. The social cost-effectiveness is apparent.

The quality of clinical trials is very crucial. Although there have been an increasing number of RCTs to evaluate the efficacy of AI in interventions in the last two years, the quality of 2/3 of these existing trials has tended to be suboptimal in terms of the referenced CONSORT statement, sample size pre-estimation, randomization, and masking^25^. The current study strictly followed the principles of a qualified RCT. Our data were randomly distributed into three datasets; the sonologists were allocated to one specific type of test based on random sequence generation; all the sonologists were blinded to the videos/images, and the recruited sample size of the datasets in this study satisfied the pre-estimation. Moreover, the three-way crossover test design avoided the potential pitfall of giving an advantage to specific reading mode, dataset and expertise levels, thus perfecting comparability without confounding effects^21^. In terms of the sample size of the study, we included 709 images/videos and involved 36 sonologists which resulted in 25,524 independent data for final analysis. Of note, as an intervention trial, sonologists played a very important role in the comparisons of three reading modes for the study. Although the number of fetuses/images/videos was not large, we made a balance between the number of fetuses/images/videos and the number of sonologists to complete the study. These 36 sonologists were randomized into three reading modes stratified by their expertise and then made to cross over and read all the images/videos. Hence, the large number of sonologists and the randomized crossover design reduced potential bias by readers among the study groups, making the groups comparable and enabling subgroup analyses.

Nonetheless, there are some limitations. First, unlike in real clinical environments, where the positive-to-negative ratio is often unbalanced, the proportions of the data with anomalies were larger, as we emphasized congenital malformation detection, and normal cases were randomly selected to maintain balance with the other nine types of abnormalities. Therefore, the effectiveness might not be the same as when used in a clinical setting. As many more normal cases were randomly excluded, selection bias might have been caused which could affect AI diagnosis and therefore the efficacy of AI in assisting normal cases recognition. Specifically, we assume that it might lead to a higher false positive rate in real-time practice, and the average accuracy of AI assistance may be affected; we will continue to explore this in future real-time clinical research involving a large proportion of the consecutive normal population. Second, as the incidence of congenital intracranial malformations is very low, we have made great efforts to enable the study completed to verify the effects of different AI assistance reading modes. While investigating ten different patterns, the actual number of images/videos is not large as each pattern only had an average amount of data around 70, with the lowest being 53. This limited number may not fully represent all the various characteristics or severity of the lesion, which could potentially decrease accuracy in recognizing each pattern. We hope that in the future our model and reading patterns can be verified in larger and more realistic clinical scenarios. Third, due to the diversity of algorithms and settings, PAICS might only represent some of the DL that will emerge in the future for the assistance of congenital malformation diagnosis. The effectiveness and suitable assistance mode must be evaluated for individual platforms. Last, even though PAICS can detect most of the fetal intracranial malformations encountered on a prenatal ultrasound screening scan^9,26^, there are still some relatively rare malformations not being included in PAICS training, and the efficacy of PAICS in assisting those rare diseases has not yet been investigated.

In conclusion, our trial indicated that PAICS may improve sonologists’ performance in detecting fetal intracranial malformation from neurosonographic data both in concurrent and in second modes. PAICS was more efficient when used concurrently for all readers. Further research is warranted in real clinical settings involving a larger sample size to investigate the assisted efficacy of PAICS in detecting congenital intracranial malformations.

## Method and materials

### Study design and data collection

This was a multireader, three-way crossover, randomized controlled trial with 36 sonologists from multiple centres by using clinical routine collected images and videos (Fig. 1). It was approved by the Institutional Review Board of First Affiliated Hospital of Sun Yat-sen University (2019421) and performed according to the Helsinki declaration. All participants provided written informed consent. The study of the clinical efficacy of deep learning artificial intelligence in detecting fetal intracranial malformations in ultrasonography was registered in http://www.chictr.org.cn on 5 July 2021 (Trial Registration: ChiCTR2100048233).

Fetal neurosonographic images and videos with normal or abnormal intracranial findings were collected consecutively from The First Affiliated Hospital of Sun Yat-sen University (FAHSYSU) and Women and Children’s Hospital affiliated with Xiamen University (WCHXU) during the period from 01 January 2021 to 01 December 2022. All the images and videos met the following criteria: (1) neurosonographic data that were acquired by experts who had more than 10 years of experience in fetal anatomy scans; (2) neurosonographic data included at least one of the three reference screening planes, acquired according to the guidelines^22,27,28^; and (3) image with an integrated skull, properly magnified, without any obvious acoustic shadow and measurement calliper overlaying. Those data, such as colour Doppler ultrasound data or data that did not satisfy the inclusion criteria, were all excluded. Each data underwent quality control before the test, which was conducted by two senior sonologists (M.L. and H.X. with over 15 years of experience) and was included only when these two experts reached a consensus.

Abnormal intracranial findings in the reference screening planes were categorized into nine different patterns according to the textbook of ultrasonographic of the prenatal brain and the ISUOG Practice Guidelines^22,27,28^: (1) nonvisualization of the cavum septi pellucidi (CSP); (2) nonvisualization of the septum pellucidum (SP); (3) crescent-shaped single ventricle; (4) mild ventriculomegaly (VM); (5) severe VM; (6) nonintraventricular cyst; (7) intraventricular cyst; (8) open fourth ventricle; and (9) megacisterna magna. Thus, counting the normal pattern as well, 10 types of different patterns were included. The prenatal sonographic diagnoses of all the images and videos were confirmed by either prenatal or postnatal magnetic resonance imaging (MRI), follow-up ultrasound examination or autopsy. Ultrasound examinations were performed using machines from six different manufacturers (GE Voluson 730 Expert/E6/E8/E10 (GEHealthcare, Zipf, Austria), Samsung UGEO WS80A (Samsung Medison, Seoul, Korea), Philips IU22 (PhilipsHealthcare, Bothell, WA, USA).

### Randomization and crossover design

As shown in Fig. 1, the eligible neurosonographic images/videos were randomly grouped into three datasets (dataset 1, dataset 2, and dataset 3), where the nine types of malformation and normal patterns had balanced proportions in each dataset. These datasets were interactively read by three groups of sonologists in three reading modes (unassisted mode, concurrent mode, and second mode). The orders of the three datasets and the three reading modes were crossover designs and thus constituted three types of tests (Fig. 1).

Thirty-six sonologists with three different levels of expertise from 32 different hospitals across the nation took part in the reading tests. They were randomly classified into three groups by expertise (*n* = 12 for each group) and were randomly assigned to one type of test with the random allocation sequence generated with a computer random number generator conducted by the research assistant. The sequence was concealed until the test began. The expert group included professors with more than 10 years of experience; the competent sonologist group included attending sonologists with 5–10 years of experience; and the trainee group included residents with 2–4 years of fetal experience in fetal anatomy scans. Sonologists in the three groups had performed at least 10000, 5000, and 1000 fetal ultrasound examinations, respectively. All sonologists were blinded to the diagnoses and had not reviewed these images/videos before. To avoid carry-over effect or contamination, all datasets were presented to sonologists in random order, and orders were different for every reader.

### The computer program designed to perform the test

Specifically, for this reading test, we designed a program that could display the images/videos, along with the ten pattern options, on a personal computer screen (see Supplementary Movie 1). The applets inserted in this program also allowed offline diameter measurements. Therefore, after reviewing the images/videos, the reader selected one of the corresponding patterns. Simultaneously, the program automatically recorded the answers and the reading time for each data point. The display settings for these three reading modes were different: there was no AI reference in the unassisted mode, whereas in the concurrent mode, the images/videos with AI diagnosis were shown in parallel with the original data at the beginning of the reading; in the second mode, after reading the original images/video and making a diagnosis, the reader clicked the “next” button to view the same data with AI diagnosis and make the final diagnosis. Therefore, in the second reading mode, there were two sets of answers for each data point, the answers before and after the AI assistance. Before implementation, all sonologists received full training on the usage of the software and only when they were qualified to use AI could they participate in the test. The PAICS was built based on a real-time convolutional neural network (CNN) algorithm, You Only Look Once, version 3 (YOLOv3). Neurosonographic images (*n* = 43078) from normal fetuses (*n* = 13400) and fetuses with CNS malformations (*n* = 2448) at 18–40 gestational weeks were retrieved from the databases of two tertiary hospitals in China and randomly assigned (ratio, 8:1:1) to training, fine-tuning and internal validation datasets to develop and internally evaluate the PAICS. An image dataset (*n* = 812) from a third tertiary hospital was used to further externally validate the performance of the PAICS and to compare its performance with that of sonologists with different levels of expertise. The macroaverage AUC and microaverage AUC of internal validation were 0.933 (0.798–1.000) and 0.977 (0.97–0.985), respectively, and the corresponding values were 0.902 (0.816–0.989) and 0.898 (0.885–0.911) for external validation, all being comparable to those of expert sonologists (0.9 [0.778–0.99], *P* = 0.891; 0.9 [0.893–0.907], *P* = 0.788). The macro- and microaverage sensitivities of PAICS were 0.876 (0.596–0.999) and 0.959 (0.941–0.973), and the macro- and microaverage specificities were 0.99 (0.95–1.000) and 0.995 (0.993–0.997) for internal validation; the macro- and microaverage sensitivities were 0.826 (0.624–1.000) and 0.817 (0.788–0.843), and the macro- and microaverage specificities were 0.98 (0.926–1.000) and 0.98 (0.976–0.983) for external validation, respectively^15^.

### Questionnaire on subjective evaluation of the effectiveness of PAICS

At the end of the test, the sonologists completed a questionnaire on their subjective evaluation of the effectiveness of the PAICS that included several questions about whether the PAICS helped identify the ten patterns in neurosonographic data. If the answer was yes, then consideration of each of the following three questions was required: 1. the subjective value on the extent of PAICS assistance was appraised with scores from 10 to 100; 2. the benefit of PAICS assistance was owed to (1) providing the diagnosis or (2) the segments localizing the lesions; and 3. which assistant modes was preferred: (1) concurrent mode, (2) second mode. Alternatively, if the sonologists regarded that AI did not help, then they answered each of the following three considerations with one of three options (0: no; 1: yes; 2: uncertain): 1. the diagnosis made by AI was incorrect; 2. AI disrupted their diagnosis; 3. the sonologists had self-confidence in their diagnosis and therefore had no need of further assistance.

### Outcomes

The primary outcome was the average accuracy (average ACC), which assessed the scores of correct identifications of all ten patterns without prior knowledge.

The secondary outcomes included the area under the curve (AUC) of the receiver operating characteristic (ROC), diagnosis accuracy (ACC), sensitivity (SEN), specificity (SPE) for multiclass classification^9^, and the time consumption of reading.

### Sample size estimation

There were three hypotheses in our study: the average ACC of (1) concurrent mode and (2) the second mode were at least 3% higher (effect size = 3%) than that of unassisted mode, respectively, and 3) the performance of one DL-assisted mode was superior to the other, defined by a 3% difference in average ACC, although no direction was specified.

Under these conditions, the two-sided α was 0.0167 for each hypothesis (α = 0.05 divided by 3 to adjust for multiple comparisons using the Bonferroni method), and the power was 90%. If the primary outcome average ACC was set to 80% for the unassisted mode and the two DL-assisted methods increased by 3%, the required sample size was 3583 images/videos per group, and the total sample size was 10749. If the power was increased to 99%, 5908 images/videos per group, or a total of 17724, would be needed. In this study, the total sample size was much larger than estimated, which ensured that the subgroup analysis also obtained sufficient power. Subgroup analysis was performed according to the levels of expertise (experts, competent sonologists, and trainees) and different types of image patterns (ten patterns).

### Statistical analysis

The performance metrics of the multiclass classification including diagnostic ACC, SEN, SPE, and AUC with their 95% confidence intervals (CIs) were estimated by micro- and macro analysis^9,29^. Micro- and macroACCs were calculated according to the confusion matrix, which was summed up by the confusion matrix from each pattern. Therefore, these ACCs were used to evaluate the diagnostic accuracy of a certain pattern among ten. ROC curves were plotted by the sensitivity (true positive rate) versus the 1- specificity (false positive rate). Fleiss’ Kappa (FK) value was calculated to assess the diagnostic agreement between sonologists with labels. Continuous variables are presented as the mean ± standard deviation (SD) or median (interquartile range, IQR), as appropriate, and categorical variables are presented as numbers and percentages. Comparisons between three independent groups were made using ANOVA or Kruskal-Wallis test for continuous variables and the Chi-squared test for categorical variables. Comparisons between two independent groups were made using the t-test or Mann−Whitney U test for continuous variables and the Chi-squared test for categorical variables. Micro and macro AUCs between two groups were compared by Delong’s test and paired t-test, respectively. Micro and macro SENs, SPEs, ACCs between two groups were compared by chi-square test for proportions and paired t-test, respectively. McNemar’s test was used to compare the preference modes of all sonologists. Multiple comparisons were corrected by Bonferroni test. All analyses were performed using R statistical software (version 4.0.2, R Core Team, 2020)^30^, and a *P* value of less than 0.0167 was considered significant for all analyses.

### Supplementary information

Suplementary Movie 1

Supplementary Material

## Data Availability

The data that support the findings of this study are available to qualified researchers on reasonable request from the corresponding authors. Please email the corresponding author Dr. Hongning Xie at xiehn@mail.sysu.edu.cn.

## Code availability

The codes of the paper are available on reasonable request. Please email the corresponding author Dr. Hongning Xie at xiehn@mail.sysu.edu.cn.

## References

[1] Lin H (2019). Diagnostic Efficacy and Therapeutic Decision-making Capacity of an Artificial Intelligence Platform for Childhood Cataracts in Eye Clinics: A Multicentre Randomized Controlled Trial. EClin. Med..

[2] Lin L (2019). Deep learning for automated contouring of primary tumor volumes by MRI for nasopharyngeal carcinoma. Radiology.

[3] Niazi MKK, Parwani AV, Gurcan MN (2019). Digital pathology and artificial intelligence. Lancet Oncol..

[4] Gulshan V (2016). Development and Validation of a Deep Learning Algorithm for Detection of Diabetic Retinopathy in Retinal Fundus Photographs. JAMA.

[5] Wang P (2020). Effect of a deep-learning computer-aided detection system on adenoma detection during colonoscopy (CADe-DB trial): a double-blind randomised study. Lancet Gastroenterol. Hepatol.

[6] Repici A (2020). Efficacy of Real-Time Computer-Aided Detection of Colorectal Neoplasia in a Randomized Trial. Gastroenterology.

[7] Steiner DF (2018). Impact of Deep Learning Assistance on the Histopathologic Review of Lymph Nodes for Metastatic Breast Cancer. Am. J. Surg. Pathol..

[8] Park A (2019). Deep Learning-Assisted Diagnosis of Cerebral Aneurysms Using the HeadXNet Model. JAMA Netw. Open.

[9] Lin M (2022). Use of real-time artificial intelligence in detection of abnormal image patterns in standard sonographic reference planes in screening for fetal intracranial malformations. Ultrasound Obstet. Gynecol..

[10] Drukker L (2022). Real-time identification of fetal anomalies on ultrasound using artificial intelligence: what’s next?. Ultrasound Obstet. Gynecol..

[11] Yang S (2019). Performance and Reading Time of Automated Breast US with or without Computer-aided Detection. Radiology.

[12] Hsu HH (2021). Performance and reading time of lung nodule identification on multidetector CT with or without an artificial intelligence-powered computer-aided detection system. Clin. Radiol..

[13] Conant EF (2019). Improving Accuracy and Efficiency with Concurrent Use of Artificial Intelligence for Digital Breast Tomosynthesis. Radiol.: Artificial Int..

[14] Mang T (2014). CT colonography: effect of computer-aided detection of colonic polyps as a second and concurrent reader for general radiologists with moderate experience in CT colonography. Eur. Radiol..

[15] Halligan S (2011). Incremental benefit of computer-aided detection when used as a second and concurrent reader of CT colonographic data: multiobserver study. Radiology.

[16] Zheng B (2004). Detection and classification performance levels of mammographic masses under different computer-aided detection cueing environments. Acad. Radiol..

[17] Moons KG (2012). Risk prediction models: II. External validation, model updating, and impact assessment. Heart.

[18] Moons KG, Altman DG, Vergouwe Y, Royston P (2009). Prognosis and prognostic research: application and impact of prognostic models in clinical practice. BMJ.

[19] Garg AX (2005). Effects of computerized clinical decision support systems on practitioner performance and patient outcomes: a systematic review. JAMA.

[20] Toll DB, Janssen KJ, Vergouwe Y, Moons KG (2008). Validation, updating and impact of clinical prediction rules: a review. J. Clin. Epidemiol..

[21] Park SH (2023). Methods for Clinical Evaluation of Artificial Intelligence Algorithms for Medical Diagnosis. Radiology.

[22] Malinger G (2020). ISUOG Practice Guidelines (updated): sonographic examination of the fetal central nervous system. Part 1: performance of screening examination and indications for targeted neurosonography. Ultrasound Obstet. Gynecol..

[23] Morris JK (2019). Epidemiology of congenital cerebral anomalies in Europe: a multicentre, population-based EUROCAT study. Arch. Dis. Child.

[24] Population statistics, world statistical data. http://populationstat.com. (2023).

[25] Zhou Q, Chen ZH, Cao YH, Peng S (2021). Clinical impact and quality of randomized controlled trials involving interventions evaluating artificial intelligence prediction tools: a systematic review. npj Digital Med..

[26] Van den Veyver IB (2019). Prenatally diagnosed developmental abnormalities of the central nervous system and genetic syndromes: A practical review. Prenat. Diagn..

[27] Paladini D (2007). ISUOG Practice Guidelines: Sonographic examination of the fetal central nervous system: guidelines for performing the ‘basic examination’ and the ‘fetal neurosonogram’. Ultrasound Obstet. Gynecol.

[28] Timor-Tritsch IE, M. A., Pilu G., Malinger G. *Ultrasonography of the Prenatal Brain (Third edition)*, (The McGraw-Hill Companies, Inc.: Town, 2012).

[29] R, R.C.T. A language and environment for statistical computing. *R Foundation for Statistical Computing*, Vienna, Austria, (2020).

[30] Team, R. C. *R: A language and environment for statistical computing*. (Vienna, Austria. 2020).

