# Supplementary material for: Deep learning system improved detection efficacy of fetal intracranial malformations in a randomized controlled trial
Source: NPJ Digit Med. 2023 Oct 13;6:191. doi: 10.1038/s41746-023-00932-6 (PMC10575919; doi:10.1038/s41746-023-00932-6)
Supplement: Supplementary file 2 — Supplementary Material [file 41746_2023_932_MOESM2_ESM.pdf]

Supplementary Movie 1. Demonstration of the test process

A program was designed specifically for this reading test that enabled the images/videos to be displayed on the personal computer screen along with the ten pattern options. The applets inserted in this program also allowed offline diameter measurements. Therefore, after reviewing the images/videos, the reader selected one of the corresponding patterns. The display settings for these three reading modes were different: in the unassisted mode, there was no AI reference, while in the concurrent mode, the images/videos with AI diagnosis were displayed in parallel with the original data at the beginning of the reading; in the second mode, after reading the original images/videos and making a diagnosis, the reader clicked the “Next” button to view the same data with AI diagnosis and make the final diagnosis.

Supplementary Table 1. The baseline characteristics of the included cases.

Characteristics	Total （n=558）	Dataset 1 （n=185）	Dataset 2 （n=191）	Dataset 3 （n=182）	*P*
Gestational age (weeks)	23.00 (20.00, 27.00)	23.00 (21.00, 28.00)	23.00(20.50, 27.00)	23.00 (20.00, 27.00)	0.583
Maternal age (years)	30.71±4.95	30.31±4.96	30.82±4.91	30.99±5.07	0.402
Prepregnancy BMI (kg/m2)	22.05±2.94	22.16±2.93	21.66±3.07	22.37±2.772	0.064
Preterm termination, n (%)	279 (50.00)	101 (54.59)	90(47.12)	88(48.35)	0.299
Pregnancy complications, n (%)	55% (9.85)	17 (9.18)	20(10.47)	18(9.89)	0.906

Comparisons among three independent data groups were made using Kruskal-Wallis test for continuous variables and the Chi-squared test for categorical variables.

Supplementary Table 2. Performance improvements of sonologists of three expertise levels in fetal neurosonographic image patterns recognition with two DL-assisted reading modes (assessed with macroaverge).

Metrics	Expertise	Unassisted mode	Concurrent mode	Second mode	*P* values of multiple comparisons		
					Concurrent vs. Unassisted	Second vs. Unassisted	Concurrent vs. Second
AUC_{macro}	Expert	0.87 (0.74, 0.99)	0.90 (0.79, 1.00)	0.91 (0.82, 1.00)	0.001	0.001	0.024
	Competent	0.86 (0.71, 1.00)	0.90 (0.79, 1.00)	0.92 (0.81, 1.00)	0.003	0.001	0.011
	Training	0.83 (0.64, 1.00)	0.88 (0.75, 1.00)	0.88 (0.75, 1.00)	0.001	0.001	0.416
SEN_{macro}	Expert	0.77 (0.51, 1.00)	0.82 (0.60, 1.00)	0.84 (0.64, 1.00)	0.003	0.002	0.045
	Competent	0.75 (0.45, 1.00)	0.83 (0.59, 1.00)	0.85 (0.63, 1.00)	0.004	0.001	0.032
	Training	0.7 (0.31, 1.00)	0.79 (0.52, 1.00)	0.80 (0.51, 1.00)	0.003	0.001	0.475
SPE_{macro}	Expert	0.97 (0.92, 1.00)	0.98 (0.93, 1.00)	0.98 (0.94, 1.00)	0.015	0.015	0.267
	Competent	0.97 (0.933, 1.00)	0.98 (0.93, 1.00)	0.98 (0.95, 1.00)	0.07	0.007	0.343
	Training	0.97 (0.91, 1.00)	0.98 (0.93, 1.00)	0.98 (0.93, 1.00)	0.007	0.007	0.65
ACC_{macro}	Expert	0.95 (0.90, 1.00)	0.96 (0.92, 1.00)	0.97 (0.96, 0.97)	0.001	0.001	0.031
	Competent	0.95 (0.88, 1.00)	0.96 (0.92, 1.00)	0.97 (0.93, 1.00)	0.01	0.002	0.021
	Training	0.94 (0.87, 1.00)	0.96 (0.90, 1.00)	0.96 (0.91, 1.00)	0.001	0.001	0.356

Values in parentheses are 95% Confidence Interval. AUC, area under the receiver operating characteristic curve; SEN, sensitivity; SPE, specificity; ACC, accuracy. Macro AUCs,SENs, SPEs, ACCs between each two groups were compared by paired t test. Multiple comparisons were corrected by Bonferroni method.

Supplementary Table 3. Performance improvements of sonologists of three expertise levels in fetal neurosonographic image patterns recognition with two DL-assisted reading modes (assessed with microaverge).

Metrics	Expertise	Unassisted mode	Concurrent mode	Second mode	*P* values of multiple comparisons		
					Concurrent vs. Unassisted	Second vs. Unassisted	Concurrent vs. Second
AUC_{micro}	Expert	0.87 (0.86, 0.98)	0.90 (0.89, 0.90)	0.91 (0.91, 0.92)	< 0.001	< 0.001	0.007
	Competent	0.85 (0.85, 0.86)	0.90 (0.89, 0.91)	0.91 (0.91, 0.92)	< 0.001	< 0.001	0.019
	Training	0.83 (0.82, 0.84)	0.88 (0.87, 0.89)	0.88 (0.88, 0.89)	< 0.001	< 0.001	0.521
SEN_{micro}	Expert	0.76 (0.75, 0.70)	0.81 (0.80, 0.83)	0.84 (0.82, 0.85)	< 0.001	< 0.001	0.016
	Competent	0.74 (0.72, 0.75)	0.82 (0.81, 0.83)	0.84 (0.83, 0.85)	< 0.001	< 0.001	0.037
	Training	0.69 (0.67, 0.71)	0.78 (0.77, 0.80)	0.79 (0.77, 0.80)	< 0.001	< 0.001	0.582
SPE_{micro}	Expert	0.97 (0.97, 0.98)	0.98 (0.97, 0.98)	0.98 (0.98, 0.98)	0.542	< 0.001	0.004
	Competent	0.97 (0.97, 0.97)	0.98 (0.97, 0.98)	0.98 (0.98, 0.98)	< 0.001	0.359	0.008
	Training	0.97 (0.96, 0.97)	0.98 (0.97, 0.98)	0.98 (0.97, 0.98)	1.000	0.069	0.069
ACC_{micro}	Expert	0.95 (0.95, 0.96)	0.96 (0.96, 0.97)	0.97 (0.96, 0.97)	0.260	<0.001	0.009
	Competent	0.95 (0.94, 0.95)	0.96 (0.96, 0.97)	0.97 (0.98, 0.99)	0.187	0.002	0.021
	Training	0.938 (0.93, 0.94)	0.96 (0.95, 0.96)	0.96 (0.95, 0.96)	0.192	0.004	0.134

Values in parentheses are 95% CI. AUC, area under the receiver operating characteristic curve; SEN, sensitivity; SPE, specificity; ACC, accuracy. Micro AUCs between each two groups were compared by Delong's test. Micro SENs, SPEs, ACCs between each two groups were compared by chi-square test for proportions. Multiple comparisons were corrected by Bonferroni method.

Supplementary Table 4. The time consumption comparisons between three different reading modes

	Unassisted mode	Concurrent mode	Second mode	*P* value for multiple comparisons		
				Concurrent vs. Unassisted	Second vs. Unassisted	Concurrent vs. Second
All	6.00 (4.00, 15.00)	7.00 (4.00, 16.00)	11.00 (7.00, 22.00)	< 0.001	< 0.001	< 0.001
Training	6.00 (3.00, 15.00)	7.00 (4.00, 16.00)	12.00 (7.00, 23.00)	< 0.001	< 0.001	< 0.001
Competent	6.00 (3.00, 15.00)	7.00 (4.00, 16.00)	10.00 (7.00, 21.00)	< 0.001	< 0.001	< 0.001
Expert	7.00 (4.00, 14.00)	8.00 (4.00, 18.00)	11.00 (7.00, 22.00)	< 0.001	< 0.001	< 0.001

Mann−Whitney U test was applied for the comparisons of the time consumption between each two reading modes. Multiple comparisons were corrected by Bonferroni method.

Supplementary Table 5. The subjective evaluation of sonologists on the efficacy of DL-aided detection.

Questionnaire items		All	Training	Competent	Expert	*P*
Evaluation scores		80 (70, 80)	70 (70, 80)	80 (70, 80)	80 (70, 80)	0.594
Improvement Reasons	Diagnosis (n, %)	28 (77.8)	9 (75.0)	9 (75.0)	10 (83.3)	0.852
	localization (n, %)	33 (91.7)	11 (91.7)	10 (83.3)	12 (100.0)	0.336
Preferred assisted mode	Concurrent mode (n, %)	22 (61.1)	7 (58.3)	7 (58.3)	8 (66.7)	0.890
	Second mode (n, %)	14 (38.9)	5 (41.7)	5 (41.7)	4 (33.3)	0.890

Comparisons among three sonologists groups were made using Kruskal-Wallis test for continuous variables and the Chi-squared test for categorical variables. No multiple comparison was made.
